# How visiting nurses detect symptoms of disease progression in patients with chronic heart failure

**DOI:** 10.1080/17482631.2020.1735768

**Published:** 2020-03-01

**Authors:** Chinatsu Taniguchi, Ayako Okada, Natsuko Seto, Yasuko Shimizu

**Affiliations:** a School of Nursing, Mukogawa Women’s University, Hyogo, Japan; b Division of Health Sciences, Osaka University Graduate School of Medicine, Osaka, Japan; c Faculty of Nursing, Japanese Red Cross College of Nursing, Tokyo, Japan; d Faculty of Nursing/Graduate School of Nursing, Kansai Medical University, Osaka, Japan

**Keywords:** Nursing assessments, symptom monitoring, chronic heart failure, chronic disease, heart failure, home care, qualitative synthesis method, KJ method

## Abstract

**Purpose**: Chronic heart failure patients are often rehospitalized because they fail to seek appropriate, timely care. However, both patients and nurses experience difficulties in detecting signs of heart failure exacerbation. We aimed to qualitatively investigate how visiting nurses detect symptoms of disease progression in chronic heart failure patients in their homes.

**Methods**: Participants were three experienced home-visit chronic heart failure nurses who completed one-to-one interviews. Data were analysed using the qualitative synthesis method (KJ method).

**Results**: Six themes were identified that reflected detection of disease exacerbation and nursing support to prevent disease progression: difficulty of judging deterioration in patients with comorbidities; ascertaining conditions needing immediate intervention; detection of illness progression from changes in the patient’s appearance; inferring instability in physical condition from patients’ concerns and questions; arranging to ensure continued management of the patient post discharge; and instructing patients to ensure they never return to their old habits.

**Conclusions**: The findings indicate that nurses experience challenges in detecting illness progression and judging when outpatient or hospital care is needed. However, nurses use a range of signs and symptoms to determine deterioration. Home-visit nurses thus play a crucial role in the post-hospital care of chronic heart failure patients.

## Introduction

It has been estimated that the number of heart failure patients in Japan will reach 1.3 million by 2030 (Okura et al., 2008) and a rising trend has been predicted that reflects the overall ageing of society. The rehospitalization rate for chronic heart failure patients in Japan is 27% within 6 months and 35% within 1 year (Miyuki Tsuchihashi-Makaya et al., 2009); this high rehospitalization rate is comparable to that of Western countries (Bergethon et al., 2016; Lund & Savarese, 2017). In addition, because of a trend towards shorter hospital stays, chronic heart failure patients have increasing home medical care and treatment needs.

Although hospitalization owing to the onset of acute heart failure permits inpatient treatment (which improves the patient’s condition and haemodynamics), myocardial function does not fully recover but gradually deteriorates. The resulting cycle of hospitalization and rehospitalization for exacerbation of heart failure has a negative impact on life prognosis (Gheorghiade et al., 2005). Therefore, the use of early detection and treatment to avoid hospitalization for acute exacerbation is key to improving patients’ long-term quality of life.

One factor that triggers the rehospitalization of chronic heart failure patients is patients’ failure to pursue medical care at the appropriate time (Goldberg et al., 2008; Evangelista, Dracup, & Doering, 2000; Patel, Shafazand, Schaufelberger, & Ekman, 2007). There is evidence that delays in seeking care limit patients’ ability to recognize, interpret, and evaluate their heart failure condition (Gravely-Witte, Jurgens, Tamim, & Grace, 2010). Although patients can recognize deterioration in their condition, it is difficult to differentiate between conditions that require treatment and conditions that resolve themselves with time. Thus, recognition of deterioration is not necessarily linked to the pursuit of medical care. This leads to delays in early detection and treatment.

There has been much research on telemonitoring and remote monitoring of health conditions that indicates the possibility of remote monitoring for electrocardiograms, weight, and blood pressure. Home management of heart failure patients is also possible by relaying vital signs to appropriately trained medical staff; this can improve heart failure patients’ quality of life through the collation of appropriate information about their illness. However, there is no consensus on whether home monitoring leads to improvements in diagnosis (Martínez et al., 2006). One study (Jaana, Sherrard, & Paré, 2019) reported that telemonitoring improved older people’s confidence in their ability to evaluate and address heart failure symptoms. However, it also decreased their ability to take part in the decision-making process about their condition. It is difficult for patients to identify and manage early symptoms of worsening heart failure. Survey research on unexpected transfers of heart failure patients from a long-term care setting to an acute-phase hospital suggests that nurses find it difficult to detect deteriorations in patients’ condition and to judge acute exacerbation of the condition (Strachan et al., 2014). Furthermore, it is difficult for medical professionals to judge exacerbation of heart failure in patients suffering simultaneously from multiple illnesses (Faxon et al., 2004). For clinicians, early detection of worsening heart failure is an important issue for disease management. Home-care strategies using telemonitoring through electronic implantable cardiac devices to measure haemodynamics are also being tried (Karamichalakis et al., 2018). Detecting deterioration in heart failure is difficult for both patients and nurses.

In recent findings, frailty is a predictor for hospitalization after the diagnosis of heart failure (Bottle et al., 2019). Therefore, there may be an atypical symptom that is also an early sign of worsening symptoms. To address this issue, this study focused on visiting nurses, who provide patients with long-term nursing and therefore play a key role in patients’ everyday lives. We thought that it was possible that visiting nurses would recognize the process of worsening heart failure in their patients. We examined whether nurses observed particular symptoms during home visits to chronic heart failure patients before hospitalization, and explored their attempts to connect their activities with early detection and treatment of disease progression. As far as we know, it is not clear how visiting nurses detect worsening symptoms of heart failure, and this information may be useful for future home-visit nursing. The purpose of this study was therefore to clarify how visiting nurses detect symptoms of disease progression in chronic heart failure patients in their homes.

## Methods

### Design

This study employed a descriptive qualitative design and used individual semi-structured interviews. The qualitative synthesis method (KJ method) was used to explore the experience of visiting nurses in detecting symptoms of disease progression.

### Participants

In 2011, the Japan Nursing Association began to accredit the education of nurses certified to treat chronic heart failure. However, nurses specialized in chronic heart failure are not yet popular in the home-care field. Visiting nurses treat people suffering from a wide range of ailments, not just chronic heart failure. Therefore, this study selected visiting nurses with 3 years or more experience of home care of chronic heart failure patients. The supervisors of visiting nurse facilities were asked to recommend candidate participants for this study. All candidates were selected as participants. The aims and procedures of the research were explained both orally and in writing to all study participants, who provided their written consent.

### Data collection

Interviews were carried out in October and November 2013. The interviews were conducted in closed rooms at the participants’ workplaces or in other locations where others would not overhear them. One-to-one interviews were conducted by the author who was a female and had abundant experience in interviews. The timing was arranged at participants’ convenience. Each interview lasted between 30 min and 1 h. The interview guide included a question about whether the patient exhibited any apparent conditions or symptoms before hospitalization. Interviews were with participants’ permission and verbatim transcripts of these used as data.

### Analytical methods

We used the qualitative synthesis method (KJ method) originally developed by Jiro Kawakita to analyse the data (Kawakita, 1967, 1970; Yamaura, 2008). This method is a type of qualitative inductive analysis used to extract meaning and essence from a random situation. It permits the structural expression of data, without abstracting the many elements found in the phenomena. The qualitative synthesis method (KJ method) has a wide range of applications in education, industry, and local affairs and government (Fukuda, Shimizu, & Seto, 2015). In recent years, it has been used in nursing to capture the prevailing circumstances in random clinical contexts, consider the essence of the problem, and derive pathways towards solutions. In this study, the qualitative synthesis method (KJ method) was useful in capturing the prevailing circumstances in the detection of symptoms of disease progression of chronic heart failure patients in the context of home-visit nursing. We believe that this method could inform future care provision.

There are three stages to analysis using the qualitative synthesis method (KJ method): code making, grouping, and chart making (Kawakita, 1967, 1970; Yamaura, 2008). First, we conducting code making. Focusing on how visiting nurses detected the symptoms of disease progression in chronic heart failure patients, the verbatim transcripts generated in the interviews were used as data. These data were unitized so that one code was assigned to one piece of semantic content (central assertion). During the code-making process, care was taken to preserve the integrity of participants’ statements. Next, grouping was performed. The codes created for the unitized data were laid out in an expanded format for easy readability. The expanded-format units of code were read through repeatedly and codes whose central assertions were similar were grouped on two to three sheets. After the initial grouping was finished, the essence of the multiple codes in the group was expressed in a short summary. In the next grouping, the short summaries generated were used as code units symbolizing their particular group. Grouping was repeated in the same way until five to six sheets of code unit had been produced. Finally, the code units of the final group were spatially arranged in a figure and chart making was performed. When constructing the arrangement, the logical relationships between the code units in the final group were explored and the resulting structure was visualized. The essence of the content of the final group’s code units was aggregated and abbreviated in a short summary. The analysis was carried out by collating all the visiting nurse interview data from the verbatim records and encoding it.

### Ensuring credibility and authenticity

The researchers who conducted the analysis had taken part in the training programmes on the qualitative synthesis method (KJ method) and were experienced in using it. The analysis process was monitored by two supervisors who were experts in the KJ method. Therefore, we made every effort to ensure the credibility and authenticity of the findings.

### Ethical considerations

This study was conducted with the approval of the regional Research Ethics Committee. The research aims and procedures were explained both orally and in writing to all candidate study participants when their consent to participation was sought. Participants were informed that participation was voluntary; that they had the choice of refusing to participate or withdrawing at any time; that the interview data would be stored to ensure anonymity of individual participants; that the findings could be disclosed; that the researchers would keep all data under conditions of strict confidentiality until the end of the study; and that the data would be disposed of in a responsible manner at the end of the study.

## Results

### Participants and interview results

Three people participated, all female. Participants were between 30 and 50 years old and worked for different visiting nursing stations. Participants had 4 to 10 years of experience as visiting nurses and had 3 to 10 years of experience caring for chronic heart failure patients. The average length of the interviews was 33 min.

How do visiting nurses detect the symptoms of disease progression in chronic heart failure patients?

**Figure 1.**
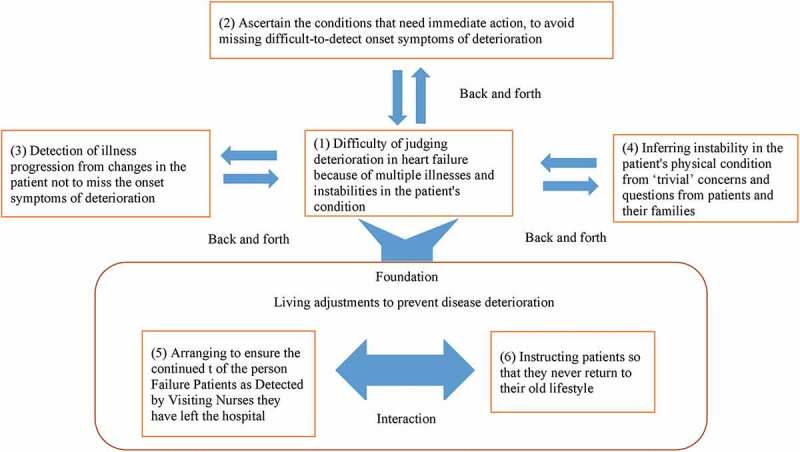
How visiting nurses detect symptoms of disease progression in patients with chronic heart failure: Identified Themes

The results of the interview data 40 were finally aggregated into six code units. Figure 1 shows the structure of the relationships between the units. The relationships that emerged between these code units with reference to how visiting nurses detected the symptoms of disease progression in chronic heart failure patients are described below.

Visiting nurses faced a challenging situation in detecting the symptoms of disease progression in chronic heart failure patients. They found it was difficult to judge deterioration in the heart because patients often had comorbidities (1). However, visiting nurses did all they could to identify conditions that required immediate action and avoid missing the difficult-to-detect symptoms of onset of deterioration (2). Nurses found that medical readings did not help them to detect these symptoms of deterioration in heart failure. Instead, they determined illness progression from observing changes in the patient’s appearance (3). They also inferred instability in patients’ physical condition from apparently trivial concerns and questions from patients and their families (4). It was not possible to judge the patient’s condition from slight changes in vital signs and overall condition. These processes embodied two types of nursing support that form the foundation of the prevention of disease progression and are undertaken daily. The first is arranging to enable the continued management of the person’s physical condition in a way that suits them after they have left the hospital (5), and the second is providing information to patients so that they never return to their old lifestyle (6).

(1) Difficulty of judging deterioration in heart failure because of multiple illnesses and instabilities in the patient’s condition

This code unit concerns the process of detecting the symptoms of heart failure deterioration and reflects the problematic situations encountered by visiting nurses caring for chronic heart failure patients. In the home environment, most patients are elderly and have multiple illnesses and sometimes it is difficult to judge whether their condition stems from heart failure or from some other illness or illnesses. In addition, for patients whose condition is unstable, there may be no need for hospitalization by emergency transportation, even in cases where symptoms appear. These factors characterize a background in which it is difficult for visiting nurses to judge heart failure deterioration.

(2) Ascertain the conditions that need immediate action, to avoid missing difficult-to-detect onset symptoms of deterioration

As shown in the quotes below, visiting nurses made every effort to closely observe onset conditions of deterioration in heart failure that were not yet clearly understood as such. They attempted on an experiential basis to ascertain from the patient’s condition which symptoms should be dealt with first.

Even though there was no oedema that would indicate the onset of heart failure, and there seemed to be no problem in the patient’s breathing, it seemed to me that the way they were breathing sounded a little bit rougher than usual. (E-1-8)

The thing is, on the other hand you have people with heart failure getting put into hospital because they’re dehydrated, right? […] But, you know, they didn’t have any swelling at all, not at all until the day before [hospitalization], and they weren’t swollen at all the day before, either. If anything, they were dried up. (B-29)

Well, at the end of the day what stood out as different is the way the saturation was down, you see? I didn’t think there was anything particularly out of the ordinary about the blood pressure or anything. […] If you were waiting [for a response] and went to [the] visiting nursing [station] you would end up calling for emergency services or something. (B-20)

(3) Detection of illness progression from changes in the patient’s appearance rather than from medical readings

This code unit clarified particular aspects of home-visit nursing. Typically, visiting nurses detected disease progression not from symptoms of heart failure or numerical readings of vital signs, but rather from aspects of their interactions with individual patients in their daily lives (e.g., noticing that the patient looked somehow different from usual).

When they get really exhausted, even neat people piss themselves and let it go, and just throw their adult diapers on the floor beside the bed. (E-1-16)

Anytime you go—you’re a visiting nurse and all—anyway, they’ll have a cup of tea ready for you. They’ll even put out their best china, coffee cups, and all. But, just when they’re going to go downhill [in terms of their condition], they don’t [get anything ready]. When you go [on a home visit], they’re just lying there snoring or whatever. They eat their meals and whatever, but otherwise they’re just dozing off and that […]. (B-55)

(4) Inferring instability in the patient’s physical condition from “trivial” concerns and questions from patients and their families

Visiting nurses noticed the possibility of disease deterioration through the slight anxieties of patients and their families before hospitalization.

Well, you see, the patient’s physical condition, when you think about it, you’re thinking, so maybe the patient’s physical condition is a bit unstable or whatever, you know? [When the patient says that] they get a cramp in the leg and what not, when they want to walk, when they get a bit excited, you wonder from this end how to take their pulse—it’s hard for us to take their pulse, and then you’re there going, ‘How are we supposed to take it easily?’ and … [there are questions]. (C-26)

The thing is, because my husband was taking more and more drugs, he ended up getting terribly nervous. […] My husband gets his meals and all that given to him, so he says things like ‘What should I do about meals?’ and ‘They don’t need to stress me out any more by giving me any more drugs or anything,’ you know? (C-22)

### Nursing support to prevent disease progression

Visiting nurses’ interventions in patients’ everyday lives to prevent disease progression were based on the two forms of nursing support described below. The results suggest that it is because these nursing support bases are in place that visiting nurses are capable of detecting the circumstances of disease progression in a multifaceted manner.

(5) Arranging to ensure the continued management of the person’s physical condition in a way that suits them after they have left the hospital

This code unit indicated that visiting nurses match the individual circumstances of chronic heart failure patients and thoroughly acquaint themselves with the patient’s everyday life circumstances through home visits once or twice a week, liaising with family and helpers on matters such as meals, everyday life, toilet practices, and drug intake.

(6) Instructing patients so that they never return to their old lifestyle

Some chronic heart failure patients gradually return to their original habits regarding factors such as activity levels and liquid intake as they try to adapt. This can result in repeated deterioration in their condition. The data indicated that visiting nurses monitor patients’ condition and urge caution to prevent sudden lifestyle changes.

## Discussion

The results of this study revealed how visiting nurses detect signs of disease in patients with chronic heart failure. Three main themes emerged regarding the detection of disease deterioration by visiting nurses. These were named as follows: Ascertain the conditions that need immediate action, to avoid missing difficult-to-detect onset symptoms of deterioration; Detection of illness progression from changes in the patient’s appearance rather than from medical readings; and inferring instability in the patient’s physical condition from “trivial” concerns and questions from patients and their families. These themes reveal how visiting nurses recognize the early symptoms of heart failure deterioration, which are difficult to identify from the symptoms and signs of general heart failure.

The first theme concerns careful observation of the physical symptoms of heart failure. One of the symptoms of general heart failure is oedema, but (depending on the patient’s condition) this may first emerge as a symptom of left heart failure such as pulmonary congestion; it is necessary for both nurses and patients to detect this symptom. It is difficult to control moisture levels and dehydration can be caused by oral administration of a diuretic. Therefore, skilful judgement of the disease condition is necessary. Linked to the issue of recognizing the signs of heart failure deterioration is the difficulty of judging deterioration because of multiple illnesses and instabilities in the patient’s condition. In developed countries, 10% of elderly people have heart failure (McMurray et al., 2012) and 39% of heart failure patients have five or more non-cardiac complications (Braunstein et al., 2003). Therefore, it is very difficult to determine whether symptoms arise from heart failure or other complications. In a home situation where medical examination is difficult, it is particularly challenging to determine whether the patient needs to go to hospital or to an outpatient department and to judge the condition of the disease.

The findings reflected the individual responses of visiting nurses in detecting illness progression from changes in the patient’s appearance rather than from medical readings, the detection of unusual changes in the patient was possible only by observing changes in the patient’s character and activity. One nurse mentioned a weakening of self-care related to excretion as an example of a sign of disease deterioration. Observation of signs such as these, which are not directly related to heart failure symptoms, may reduce the difficulty of detecting early signs of cardiac insufficiency. We believe that this finding is one of the more important aspects of this research, and suggests the need to be sensitive to changes in the patient’s appearance and behaviour that may indicate disease.

The third theme (Inferring instability in the patient’s physical condition from “trivial” concerns and questions from patients and their families) suggests that the signs of deteriorating heart failure can be recognized from complaints from patients and their families. In the absence of obvious symptoms or signs of heart failure, such complaints tend to be regarded as “indeterminate complaints” or “nervous complaints”. However, these may indicate that the patient’s symptom cognition is high and needs examining. By understanding the symptoms recognized by the patient, nurses can cooperate with patients to detect symptoms early.

Finally, visiting nurses are in a unique position to detect the signs of heart failure deterioration. This is because they are responsible for arranging to ensure the continued management of the person’s physical condition in a way that suits them after they have left the hospital. In addition, nurses can instruct patients so that they never return to their old lifestyle. A visiting nurse can detect a minor change in a patient because he or she helps the patient adjust to their living conditions so that their condition does not deteriorate. Nurses achieve this by observing the patient’s condition and individual situation daily.

This study found that visiting nurses observed not only the general symptoms of heart failure but also the symptoms and signs of the disease condition because they carefully observe the living conditions of patients. Although we focused on visiting nurses, these findings could also apply to outpatient nursing. By collaborating with visiting nurses, health-care workers can check changes in the patient’s living situation and ask for a consultation at an early stage if there are concerns. Hospitals need to understand the unique knowledge and viewpoint of visiting nurses. Nurses may be hesitant to report “trivial” signs of heart failure deterioration. However, to identify and treat the early signs of heart failure deterioration, medical personnel need to collaborate and understand each other’s perspectives. In other words, the availability of devices is limited in home-visit nursing care. Therefore, in addition to general symptoms of heart failure deterioration, worsening of symptoms is judged from the patient’s condition and minor changes in their life. Because this judgement includes uncertain elements, it is necessary to acquire objective data from examinations in clinics and hospital outpatient departments to corroborate the judgements of visiting nurses. In Japan, meetings are held between hospitals and home-care staff before discharge, but there are issues with communication after discharge. Ongoing cooperation would be ideal. This should be supported by information from other staff, such as helpers and physical therapists involved in home care. It would be helpful to train visiting nurses to communicate smoothly with staff in other roles. Research on care delays has mainly focused on hospitalization and emergency services (Gravely et al., 2011), with most studies examining in-hospital care. Future studies should consider the importance of home care.

## Limitations

In this study, we targeted three nurses who had at least 3 years of work experience in home care for patients with chronic heart failure. The findings may therefore not reflect the diversity of experiences in detecting the signs of heart failure deterioration and cannot be generalized. However, we believe that our detailed interviews clarified essential elements of how nurses perceived the signs of heart failure deterioration in actual nursing practice, which is influenced by complex and diverse factors.

The participants were shown the results of the study but did not provide any feedback. To improve the quality of research, it would be helpful to confirm the findings with the participants and reflect their opinions.

## Conclusion

It is difficult for patients with chronic heart failure to detect signs of heart failure deterioration. This study qualitatively surveyed home-visit nurses’ experiences in detecting disease deterioration in patients with chronic heart failure. Six themes were identified that reflected detection of disease exacerbation and nursing support to prevent disease progression. In addition, this study found that visiting nurses observed the symptoms and signs of disease condition as well as the general symptoms of heart failure because they carefully monitored their patients’ living conditions. However, it remains difficult to judge early signs of worsening symptoms in the context of multiple diseases and unstable medical conditions. Therefore, visiting nurses and clinics or hospital outpatient departments need to collaborate and understand each other’s perspectives.
